# Book Reviews

**Published:** 1917-04

**Authors:** 


### BOOK REVIEWS

Books mentioned may be inspected at and ordered through this office. So far as possible, books received in any month will be reviewed in the issue of the second month following. Pamphlets, quarterly and similar periodicals, reports, transactions, etc., will, as a rule, merely be mentioned.

---

**A Reference Handbook of the Medical Sciences**; Embracing the entire range of Scientific and Practical Medicine and Allied Science. By various writers. Third Edition, Completely Revised and Rewritten. Edited by Thomas Lathrop Stedman, A. M., M. D. Complete in eight imperial quarto volumes. Volume VII, 998 double column pages, illustrated by numerous cromolithographs and four hundred and sixty-nine half-tone and wood engravings.

Our readers will be pleased to know that this monumental work is nearing completion, Vol. 8 being already partly in type. This is the third edition, the first having appeared late in the 80's. The earlier editions have, however, been used merely to suggest the arrangement and approximate proportionate bulk of matter in different volumes as entire sub-branches of medicine have been developed since the first edition and as nearly all phases of the subject have required treatment from the standpoint of modern investigation. We need not dwell on the magnitude of the Handbook but, from personal knowledge, we think it worth while to pay a tribute to the chief editor, Dr. Thomas Lathrop Stedman. Obviously, a work of this nature would overtax not only the time but the special information possible to any single author but the critical knowledge of details possessed by this man has really enabled him to act as an editor in the true sense of the word and not merely as an executive in charge of the arrangement of the work of others.

**Materia Medica and Therapeutics,** including Pharmacy and Pharmacology; Reynold Webb Wilcox, M. A., M. D., LL.D., D. C. L., New York; P. Blakiston's Son & Co., Philadelphia. 9th edition, revised in accordance with the U. S. Pharmacopoeia IX. 860 pages, $3.50.

This is divided into two parts, I. Materia Medica and Pharmacy, II. Pharmacology and Therapeutics. Part I includes the usual preliminary consideration of definition and pharmacy, and the general divisions of the inorganic and organic materia medica. The former is discussed according to chemic classification, the latter is divided into synthetics, and drugs of vegetable and animal origin respectively. Drugs of vegetable and animal origin are subdivided according to principal physiologic actions. An appendix to this part gives the botanic and zoologic classification of drugs. Part II includes definitions, modes of administration, posology and discusses the relation between chemic constitution and physiologic action and the theories of ions and osmotic pressure. Under 16 general divisions, the action of drugs is then considered according to a logical physiologic classification. The final division, "Drugs which have no marked therapeutic properties" is, in a sense, disappointing. It deals with flavoring and coloring substances, etc. We rather hoped for a polemic attacking a considerable number of drugs well known by name and which justify considerable therapeutic nihilism. The volume is concluded with an index by disease names, suggesting reference to drugs that might be available.

---

**The Breast: Its Anomalies, Its Diseases and Their Treatment.** John B. Deaver, M. D., LL.D., Sc. D. and Joseph McFarland, M. D., Sc. D., assisted by J. Leon Herman, B. S., M. D., all of Philadelphia. P. Blakiston's Son & Co., Philadelphia. 8 colored plates, 227 illustrations, 724 pages, $9.

People generally, perhaps even the medical profession, think of the breast as a female organ of lactation, subject to a few pathologic disturbances and affording some diagnostic signs in connection with pregnancy, and liable to be affected with cancer—or rarely with some other process which is practically to be considered and treated as cancer—later in life. Even those who realize that this is a very imperfect conception, will probably be surprised at the large volume issued without padding, and the numerous conditions to which the breast is subject. Even from the practical standpoint, the breast is not to be considered as exclusively a female organ. Gynaecomastia is a large and interesting subject but the male organ quite often gives trouble and requires intelligent treatment. Supernumerary mammae and mamillae are frequently observed and are considered at length in this book. In glancing over the reference list we are ashamed to be found missing—not from any sense of wounded pride but simply because after having observed and noted these conditions in physical examinations for many years, we have never had the energy to collate and publish the notes. It is not at all likely that our own observations have been of unusual value and we mention the matter to illustrate the fact that if the profession generally noted such anomalies and reported them, the available literature would be on a far larger scale than at present. The notes on comparative physiology and anatomy are interesting but not extensive.

We would urge that this book be generally read, especially the parts dealing with the hypertrophies, non-suppurative infections and non-cancerous tumors and various other conditions with which the profession is not so familiar as it should be.

---

**A Brief Report on Mental Defectives in Buffalo and Vicinity.** Pamphlet comprising a report to the Vassar Club by a committee of its members.

This is a study, largely along lines of heredity, necessarily very incomplete but suggestive of the need of thorough study of the problem and of appropriate provision for these unfortunates. It is commended to the profession generally and especially to neurologists and those interested in medical sociology. The Chairman of the Committee is Mrs. Lucien Howe of Buffalo.

---

**The University of Buffalo Bulletin. Dept. of Arts and Sciences.** Published quarterly. The Jan. 1917 issue is No. 1 of Vol. 5.

This serves to call attention to the fact that while the University is still in need of large funds and is not yet in a position to offer a full college course, it is a condition and not a theory; that it is conducting on an adequate basis, the first two years of a college course and has progressed to a considerable degree toward the object of providing higher education in Buffalo.

---

**Report From the Department of Pathology and the Department of Clinical Psychiatry.** Central Indian Hospital for the Insane, 1913-14 and 1914-15, Vol. 6.

This is issued under the superintendency of Dr. Geo. F. Edenharter, (Indianapolis), and contains not only the usual executive and statistic reports but scientific studies of cases of great value.

---

**Clinical and Laboratory Technic.** H. L. McNeil, A. B., M. D., Galveston. C. V. Mosby Co., St. Louis. 88 pages illustrated, $1.

This is a brief, practical treatise including history taking and physical examinations, and routine laboratory methods for urine, blood, sputum, stomach contents, etc. Considerable attention is given to the Wassermann and gonoccus fixation tests. The book is valuable especially for what it omits in the way of methods beyond the time and ability of the practitioner. It is a codification of what the physician may do and really ought to do himself if he would be thorough up to the practical limit.

---

**Practical Uranalysis.** B. G. R. Williams, Director of the Wabash Valley Research Laboratory. C. V. Mosby Co., St. Louis, 142 pages, illustrated, $1.25.

After discussing the general properties of normal urine, the work is divided into chemical (qualitative), quantitative, microscopic and bacteriologic methods. It is eminently practical and written especially for the physician who wishes actually to put theories into operation. The discussion of indicans, other coloring matters and chromogens is especially to be commended and the warnings against diagnosing rare conditions, not yet thoroughly understood, should be heeded.

---

**Little Lessons in Scientific Eating.** Eugene Christian, President of the Corrective Eating Society, 450-460 Fourth Ave., N. Y. Published by the Society. Fasciculus of 24 lessons, each separately bound as a pamphlet. Price not stated.

The author is to some degree a vegetarian, that is, while recognizing milk, eggs, butter, etc., as proper foods, he would exclude meats. The lessons are intended for lay guidance and, consequently can not fulfill to the degree that many would require of the definition, the term "scientific." Waiving the question as to vegetarianism, the lessons seem to present as well as practical, under these circumstances, the general principles of dietetics and while it must be admitted that no propaganda addressed to the laity can satisfactorily solve the problem of adequate, economic and hygienic diet, without invividualized guidance, it seems to us that these lessons will go a long way toward correcting the present unintelligent use of foods by the people at large.

